# Introduction: Young Fatherhood: Lived experiences and policy challenges

**DOI:** 10.1017/S1474746415000536

**Published:** 2016-01

**Authors:** Bren Neale

**Affiliations:** FLaG Research Centre, School of Sociology and Social Policy, University of Leeds E-mail: b.neale@leeds.ac.uk

## Abstract

The entry of young people into early parenthood has long been regarded as an issue for social policy and for professional practice in the UK and internationally. Despite a steadily falling trend, most notably since 1998, the UK still has one of the highest rates of teenage pregnancy in Europe, concentrated in the most socially disadvantaged areas of the country (Office for National Statistics, 2015). The majority of these pregnancies are unplanned, with about half resulting in the birth of a child, although the extent to which this should be a cause for concern is a contested issue (Duncan *et al.*, 2010). Considerable research evidence exists on the experiences of young mothers, with a range of interventions designed to meet their needs. However, young fathers (defined as those under the age of 25, a quarter of whom are estimated to be in their teens) have, until recently, been neglected in both research and policy. Over the past decade, small pockets of research evidence on the circumstances, practices and values of young fathers have begun to coalesce into a fledgling evidence base. However, the notion of ‘feckless’ young men, who are assumed to be absent, or disinterested in ‘being there’, or, worse, regarded as a potential risk to their children, continues to hold sway, particularly in popular media and some political discourses (Neale and Davies, 2015).

The entry of young people into early parenthood has long been regarded as an issue for social policy and for professional practice in the UK and internationally. Despite a steadily falling trend, most notably since 1998, the UK still has one of the highest rates of teenage pregnancy in Europe, concentrated in the most socially disadvantaged areas of the country (Office for National Statistics, 2015). The majority of these pregnancies are unplanned, with about half resulting in the birth of a child, although the extent to which this should be a cause for concern is a contested issue (Duncan *et al.*, 2010). Considerable research evidence exists on the experiences of young mothers, with a range of interventions designed to meet their needs. However, young fathers (defined as those under the age of 25, a quarter of whom are estimated to be in their teens) have, until recently, been neglected in both research and policy. Over the past decade, small pockets of research evidence on the circumstances, practices and values of young fathers have begun to coalesce into a fledgling evidence base. However, the notion of ‘feckless’ young men, who are assumed to be absent, or disinterested in ‘being there’, or, worse, regarded as a potential risk to their children, continues to hold sway, particularly in popular media and some political discourses (Neale and Davies, 2015).

## Young parents: ‘new’ adults?

Before turning to the policy context for this themed section, it is worth considering why the idea of young fatherhood (and young parenthood more generally) evokes such moral panic and condemnation in the popular imagination. Of the many challenges facing young parents, perhaps the foundational issue relates to their youthful and dependent status. Transitions into adulthood in contemporary Britain are acknowledged to be increasingly fluid, varied and difficult to navigate (Furlong and Cartmel, 2007). Youth as a life course category is said to be expanding in a way that blurs the boundaries between youth and independent adult life. In the 1950s, as post-war employment opportunities flourished, the key life course trajectories relating to age, employment, family and household were bound together for young people as part of a new, normatively prescribed pathway into adulthood. The orderly progression involved leaving school or college, finding work, entering a stable relationship, setting up an independent marital home and starting a new family. Time spans for these transitions were also prescribed, with progress expected to occur over a decade or so, starting at the point of leaving full time education.

However, for many contemporary young people these pathways have been disrupted (McDowell, 2014). In 1975, over 60 per cent of young people moved straight from school to work at the age of sixteen, but by the mid 1990s the percentage had dropped to less than 20 per cent (Coles, 2000). The collapse of the youth labour market in the 1980s led to a steep rise in youth unemployment, an expansion of youth training schemes and, by the 1990s, an expansion in post-sixteen education and routes into further and higher education. By the year 2000, nearly 80 per cent of young people were extending their education beyond the traditional school leaving age (Coles, 2000), a trend that has been reflected and reinforced through successive legislation (the latest change, in 2015, requires young people born after September 1997 to stay in education or training until they are eighteen). Disadvantaged young people are more likely than others to experience insecure working lives, with ‘transitions characterised by flux and rapid movement around different economic statuses (‘unemployed’, ‘in training’, ‘in education’, ‘employed’) (MacDonald, 2013: 2).

These changing routes from education and training into employment have been accompanied by rising housing costs and widespread transformations in normative family practices, a substantial loosening of marital ties, and a slow but discernible uncoupling of partnering, cohabitation and parenting as the basis for family life. Of key importance here is the gradual rise of the ideology of ‘new fatherhood’: an appreciation of fathering that is grounded in the quality of parent‒child relationships, rather than the extent of contact, or the co-resident or partnership status of the parents (Smart and Neale, 1999).

These far reaching changes in life course trajectories have substantially unravelled the post war, normatively prescribed transition to adulthood. The extended trajectory from compulsory education into employment for most young people brings parallel delays and variations in the transition from dependent to independent living, with a growing pattern of nomadic young people living in temporary forms of accommodation in early adulthood. The key trajectories relating to age, family, employment and home, then, are no longer necessarily synchronised, or intertwined; they may or may not unfold in chronological order, in a linear direction, or at a uniform pace, indeed some elements of the transition may be extended while others have accelerated (Bynner, 2007; Neale, 2015): ‘[F]ar more common are complex and contradictory patterns in which the achievement of independence in one sphere of life may well involve compromises in others’ (Allen and Crow, 2001: 36). As Bynner observes, there is a need to ‘move from blanket categorisations of individuals in terms of stages bound by chronological age towards a broader conception based on a range of trajectories’ (2007: 378).

Young parents occupy a unique place in these processes. In 1981, around 50 per cent of teenage conceptions occurred within marriage, but the delayed trajectories and changing mores of family formation mean that only a small minority of conceptions now take place as part of a planned entry into parenthood within a stable relationship. It is also the case that values surrounding transitions into parenthood vary across social groups; a close layering of the generations, for example, is a more commonly occurring and acceptable pattern within disadvantaged communities (Emmel and Hughes, 2014). Viewed historically, it is not so much that young people are having children at an earlier age, it is more the case that other elements of the transition to ‘full’ adult status are occurring later in the life course. Indeed, young parents could be said to be pioneers of a ‘new’ adulthood (Wyn, 2014), one that does not rely on the idea of a prescribed and wholesale transition to a ‘finished’ and fully independent adult state at the point where all the pieces of the jigsaw fall neatly into place. Indeed, in the current climate of austerity and wholesale changes in labour markets, a secure transition into such a state may be beyond the reach of many disadvantaged young people. In this shifting historical picture, young people in their teenage years, who enter parenthood without skills, employment, resources, stable relationships or stable independent homes represent the ultimate confounding of the post-war normative path to adulthood. It is this confounding of normative ideas, coupled with evidence for a strong correlation between early conception and indices of social deprivation, that has had a profound influence on public and policy perceptions of young parents and, in some government and media circles, created such opprobrium towards them (Swann *et al.*, 2003; Ingham, 2005; Arai, 2009).

## The policy context

The shifting normative picture outlined above has given rise to a range of policy concerns about young parenthood. In the last decade of the twentieth century, Conservative policy was framed by a moralistic concern to reduce teenage pregnancy rates. This was operationalised as a policy target within the five year Health of the Nation initiative (1992‒7), although it proved to be largely ineffective (Department of Health, 1998; Arai, 2009). Under New Labour's ten-year Teenage Pregnancy Strategy (TPS, 2000‒10) (Social Exclusion Unit, 1999), the concern about young parenthood was reframed in the less moralistic language of social exclusion: a perceived correlation between early parenthood and a range of social ills, including poverty, poor education and skills, unemployment, crime and ‘troubled’ family backgrounds. Evidence on the backgrounds of young fathers tends to support this view (Quinton *et al*., 2002; Swann *et al*., 2003).

The then prime minister, Tony Blair, castigated the Tories for attacking teenage mothers while ignoring, ‘the damage [pregnancy] does to the education, employment and life chances of young women and girls’ (cited in Arai, 2009: 59). The TPS, which was rolled out nationally via a team of regional teenage pregnancy coordinators, responded to this perceived hazard by setting targets to substantially reduce conception rates among young women, and to increase (to 60 per cent) the number of young parents in education, employment and training (EET). However, the priority of the strategy was to tackle the ignorance that was perceived to be driving early conception, rather than the low expectations that were presumed to underpin the social disadvantages associated with young parenthood (Duncan *et al*., 2010). In the event, only a 30 per cent increase was achieved in EET rates over the ten-year period. It is worth putting this statistic in context. To expect very young mothers, driven by an imperative to care, to be in employment from the time of their child's birth was perhaps unrealistic, when survey data for the 1990s shows that, at that time, nearly 60 per cent of all first time mothers were not in employment during the first five years of their children's lives (Duncan *et al.*, 2010: 42). The strategy, then, presumed a life path that was not necessarily suited to the needs of young mothers (Harris *et al.*, 2005). Nevertheless, while the Teenage Pregnancy Strategy missed its targets by a considerable margin, some progress was made, and a useful framework was provided around which wider policy and practice responses could cohere.

There are two important observations to be made about these policy developments. Firstly, as the above discussion reveals (and as commentators frequently observe), parenthood in the TPS really meant motherhood: young fathers are largely invisible in the equation. Where they are referred to, it is their responsibility as financial providers or their indirect support for mothers that is stressed, rather than any direct caring role:

Young men are half of the problem and the solution . . . Young men . . . need to be targeted with information about the consequences of sex and fatherhood, including their financial responsibility to support their children. Fathers of children borne to teenage mothers will therefore be pursued vigorously by the Child Support Agency to reinforce the message that, for this group, regardless of age, they are financially responsible for their children. (Social Exclusion Unit, 1999: 97)

There is a deep-rooted assumption that fathers who live apart from their children and are not in a relationship with the mother (who is usually the primary carer) are necessarily ‘absent’ and ‘feckless’, i.e. that they are uninterested in their children and do not wish to provide financially for them. For young fathers, whose children are presumed to be the product of irresponsible and casual intimacy, these assumptions are even more deeply ingrained. These young men, then, are stigmatised on two counts ‒ as ‘absent’ fathers and irresponsible youths ‒ with few material resources to bring to parenting. No account is taken here of the potential of young fathers to contribute to the direct care of their children, and no consideration is given to their own aspirations and life chances and their needs for support in their own right (Quinton *et al.*, 2002). In these important dimensions of their lives, young fathers are notably absent from the policy script.

Secondly, while the precise relationship between early parenthood and the conditions associated with social exclusion is not clearly spelt out, in New Labour rhetoric it is early parenthood itself that is seen as a social problem. It is presented as a uniformly negative experience for young parents, their children and for society as a whole, a calamity or hazard that leads to a range of social ills. Moreover, the problem is perceived to be rooted in young people's ignorance and low expectations; it is an individual problem that requires remedying through changes to their attitudes and behaviour. Despite acknowledging the links between early conception and poverty and unemployment, the wider structural issues and measures needed to tackle these were ignored in New Labour's strategy for teenage pregnancy (Kidger, 2004; Arai, 2009; Duncan *et al*., 2010).

In 2010, at the end of New Labour's term of office, plans emerged to integrate work on the prevention of early conception and support for young parents into a variety of pre-existing policies and programmes. The TPS was to be rolled out via a range of universal and targeted services: maternity and SRE (Sex and Relationship Education) provision, structured family intervention programmes (the Family Nurse Partnership for vulnerable young mothers); Children's Centres, Education and Housing support services, and the Connexions (employment) service for young people (Department for Children, Schools and Families/Department of Health, DCFS/DH, 2010). The new approach was subsequently implemented by the incoming Coalition government (Hadley, 2014). The focus continued to be on reducing the under-eighteen conception rate, with targets set within a range of new policy documents issued between 2011 and 2014 by the Departments of Health and Education (Hadley, 2014). While young fathers’ needs were more readily identified and acknowledged in the DCFS/DH strategy document, there was little attempt to follow this through into subsequent policy directives. The Department for Education guidance for Children's Centres, for example, makes provision for supporting parents and addressing issues of poverty and inequality through the coordination of skills training and referrals to job centres, but on the needs of young parents the guidance is strangely silent, while fathers are barely mentioned (Department for Education, 2013). Starting with the disbanding of the teenage pregnancy coordinators in 2010, the new strategy for teenage pregnancy was fractured and diluted across diverse agencies, resulting in patchy and un-coordinated service provision, and an over-reliance on isolated ‘local champions’ operating outside of, or on the fringes of statutory provision. A recent parliamentary enquiry into parenting and social mobility observes that:

The present parenting support offer across the UK is fragmented, with little leadership from national government. With family policy spread across a number of departments, a lack of joined up government is a key barrier to any successful parenting support . . . Any parenting support scheme must not be overly prescriptive, and cannot be seen by parents as a punishment if it is to be successful . . . Fathers are an important resource in early years child development . . . but are under used and often side lined when family services are developed. (All Party Parliamentary Group, 2015: 5)

The issue raised by the parliamentary enquiry concerning the nature and style of delivery of parenting support schemes is highly pertinent here, given the ongoing stigmatising of young fathers and their families. Traditional moralistic responses to young parenthood had continued to find voice during New Labour's terms of office through speeches and media reports from Conservative opposition MPs. These sought to place young parents in an assumed ‘underclass’, from large, poor, ‘broken’ and troubled families, who live on welfare at the expense of hard working tax payers, and engage in anti-social behaviour, crime and drug addiction. Indeed, Duncan Smith went on to link teenage parenting with moral and cultural breakdown, placing the children, parents and extended families beyond the pale of ‘civilised society’. He also criticised ‘ineffective remedial policies, whether they take the form of more prisons, drug rehabilitation or supporting longer and more costly lifetimes on benefits’ (*The Sunday Times*, 15 February 2009, cited in Duncan *et al*., 2010: 2‒3).

Since the time of the Coalition government, an increasingly moralising and divisive narrative has sought to distinguish responsible, deserving citizens from those who are irresponsible and un-deserving. Conservative rhetoric that decries the broken society and family breakdown and equates the rise of single parent families with a descent into poverty and ‘welfare’ dependency has intensified (Patrick, 2015). For fathers in general, and young fathers in particular, this is reflected in the distinctions drawn between ‘good’ hard-working fathers and ‘feckless’, absent fathers, and between ‘vulnerable’ single mothers and the ‘runaway’ fathers who abandon them. While legislation such as the 2014 Children and Families Act, valorises the ‘good’ father through provisions for paternal leave and flexible working practices, in public discourses ‘bad’ fathers are increasingly vilified: ‘its high time “runaway” dads were stigmatised and the full force of shame was heaped upon them’ (David Cameron, quoted in Hennessy, 2011). Policy discourses may paint young fathers in a variety of ways, but they are invariably cast as a social problem and the root of the problem is presumed to lie with them.

To go back to our starting point, this state of affairs arises in part because policy responses are not adequately grounded in a robust evidence base that takes into account the realities of young fathers’ unfolding lives. In the context of ‘whole family’ policies, Morris and Featherstone (2010: 563) observe that:

[T]he last decade represents a lost opportunity to construct family policies which engage with the complexity and diversity of the lived experiences of families and contemporary family practices. Policies have not been rooted in dialogue with vulnerable and marginalised families about their needs and the challenges they experience.

The same could equally be said for policies that impact on young fathers. In the current climate, the potential for young fathers to make a positive contribution to their children's lives, and to improve their own life chances, is only slowly being realised.

## Young fatherhood: lived experiences and policy challenges

Based on a symposium on young fatherhood, held at the Social Policy Association Annual Conference in July 2014, the contributions gathered here afford an opportunity to enhance the evidence base on young fatherhood and to give a fresh appraisal of their support needs and experiences. The articles take as a starting point the insight that young fathers are generally committed to their children and that their involvement can be beneficial for all: an unplanned child is by no means an unwanted child. However, young fathers are likely to face a raft of challenges in securing and sustaining an active role as a parent, and many will need professional help and support in doing so. The first three articles present new empirical evidence on the varied circumstances of young fathers’ lives. In the first, Neale and Davies explore young fathers’ aspirations as ‘breadwinners’, documenting their desire to provide for their families and charting their varied, circuitous and tenuous routes through education and training into employment or on to benefits. The article reveals the sheer, sustained hard work of young fathers in managing the triple burden of earning, learning and caring, dispelling any vestige of the idea that they are to be written off, en masse, as feckless (cf. also Johnson, 2015). Engaging with the ‘social problems’ perspective on early parenthood, the authors refute the view that early parenthood ‘causes’ social deprivation. They show that the fortunes of young fathers are shaped in the main by their pre-existing socio-economic circumstances, indicating the need for more robust support that begins much earlier in their EET journeys, and for greater recognition of the structural factors that shape their life chances. In the second article, Ferguson explores the varied ways in which young fathers engage with a flagship early intervention scheme: the Family‒Nurse Partnership. His evidence demonstrates the importance of moving beyond blanket categorisations of fathers, or the professionals that support them, as ‘good’ or ‘bad’. Painting a more nuanced picture, he shows how a constellation of dynamic factors operate to create distinctive interactions between fathers and practitioners. He also explores the reasons for the disengagement of highly marginalised young men, those whose deep experiences of social suffering indicate the need for sustained, therapeutic support from specialist practitioners. In the third article, Ladlow and Neale take up this theme, exploring the provision offered to highly marginalised, young offender fathers in and beyond the custodial estate. Drawing on narratives from the young men and the practitioners who work with them, the article examines and invites us to rethink the dominant framing of these young men as either a ‘risk to’, or ‘resource for’ their children, bringing ideas of redemption, agency and emergent identities over time more centrally into the picture. In her review article, Lau Clayton places the evidence from the empirical articles in a broader context, drawing on key studies to build a more definitive picture of the relationships, circumstances and support needs of young fathers, in the process revealing the heterogeneous nature of their lives. The evidence presented on the extent to which young fathers seek to engage with their children, and maintain a commitment to working constructively with the mothers, is a significant corrective to popular perceptions of these young men. The final article is practitioner-led, based on a review of service provision carried out by Cundy on behalf of a consortium of voluntary sector organisations (the Family Strategic Partnership). She provides an overview of the joint role of statutory and voluntary sector organisations in supporting young fathers, mapping out a number of good practice pathways, through maternity services, children's centres, schooling, housing and custodial settings, that enable young fathers to meet the challenges of early parenthood.

A key theme underpinning these articles, and representing a departure from much of the existing literature in this field, concerns the importance of thinking dynamically in understanding the lives of young fathers, and, indeed, the value of building a life course perspective into policy and practice responses and processes. The emerging picture here is complex. The evidence shows that tailored support, that is timely, provided at an early stage in the journey into parenthood, and sustained over time, can foster new parental identities and a renewed sense of purpose in life for young fathers, to the benefit of all. There is clearly scope for adopting a redemptionist ethos in working with highly marginalised young fathers, those whose biographies are marked by persistent levels of poverty and emotional hardships. Side-lining young men who may be deemed ‘risky’ does not address the issues they are facing, and, at the least, may do little to address safeguarding issues for their children or the mothers. In these challenging circumstances, the emphasis on specialist, sustained professional support that is tailored specifically to the needs of these young men would seem to be vital.

In the current climate, there is clearly a case for rethinking the notion that young fathers are ‘hard to reach’. It is worth recalling that this is stigmatising terminology, which places responsibility for a perceived lack of engagement with ‘hard to reach’ people themselves (Freimuth and Mettger, 1990). Perhaps it is time to turn this idea on its head, to consider how services respond and how they may be ‘hard to access’ for this client group (Hadley, 2014). The potential to meet young fathers half way, to find creative ways to enter into their world rather than assuming an engagement solely on professional terms, may be part and parcel of an effective strategy to build sustained, trusting and supportive professional relationships. Yet while the nature and quality of interactions between clients and practitioners is important, ultimately it is not simply about the responses of individual service providers (Osborn, 2015). It is also vital to address the infrastructure and service ethos within which practitioners operate, the policy directives that guide their engagement, the funding and resources available to facilitate their work and, not least, government measures to tackle the structural inequalities that create persistent hardship. These are the crucial prerequisites in supporting some of the most marginalised young men and their families in our society.
